# Improved RNA quality and TaqMan^® ^Pre-amplification method (PreAmp) to enhance expression analysis from formalin fixed paraffin embedded (FFPE) materials

**DOI:** 10.1186/1472-6750-8-10

**Published:** 2008-02-06

**Authors:** Jinghuan Li, Paul Smyth, Susanne Cahill, Karen Denning, Richard Flavin, Sinead Aherne, Marco Pirotta, Simone M Guenther, John J O'Leary, Orla Sheils

**Affiliations:** 1 Deptment of Histopathology, University of Dublin, Trinity College, Dublin, Ireland; 2 Applied Biosystems, Foster City, CA, USA

## Abstract

**Background:**

Archival formalin-fixed paraffin-embedded (FFPE) tissues represent an abundant source of clinical specimens; however their use is limited in applications involving analysis of gene expression due to RNA degradation and modification during fixation and processing. This study improved the quality of RNA extracted from FFPE by introducing a heating step into the selected extraction protocols. Further, it evaluated a novel pre-amplification system (PreAmp) designed to enhance expression analysis from tissue samples using assays with a range of amplicon size (62–164 bp).

**Results:**

Results from the Bioanalyzer and TaqMan^® ^data showed improvement of RNA quality extracted using the modified protocols from FFPE. Incubation at 70°C for 20 minutes was determined to be the best condition of those tested to disrupt cross-links while not compromising RNA integrity. TaqMan^® ^detection was influenced by master mix, amplicon size and the incorporation of a pre-amplification step. TaqMan^® ^PreAmp consistently achieved decreased C_T _values in both snap frozen and FFPE aliquots compared with no pre-amplification.

**Conclusion:**

Modification to extraction protocols has facilitated procurement of RNA that may be successfully amplified using QRT-PCR. TaqMan^® ^PreAmp system is a robust and practical solution to limited quantities of RNA from FFPE extracts.

## Background

Archival Formalin-Fixed, Paraffin-Embedded (FFPE) tissue samples represent a robust and invaluable source of human tissue for gene expression analysis. Compared to fresh and snap frozen tissue, FFPE tissue has an inherent advantage in that retrospective patient data, including survival history and treatment response etc, is readily available, allowing immediate comparison with clinical pathological parameters. Data generated can potentially highlight biomarkers useful in disease classification, diagnosis and prognosis, and potentially elucidate novel therapeutic targets [1,2].

However, these tissues have not been widely used in molecular biology due to the degradation and chemical modification of RNA extracted from FFPE blocks. RNA extracted from FFPE is degraded to fewer than 300 bases [3] in length because archived blocks are often stored at room temperature for long periods of time. The situation is made more complicated by the fact that RNA is modified by methylol groups to form cross-links with protein or nucleic acid during formalin fixation [4-6], which results in poor yields [1,7] and compromised extracts.

Real-time quantitative TaqMan^® ^reverse transcriptase-polymerase chain reaction (QRT-PCR) analysis has been introduced as a sensitive, accurate, and highly reproducible method to study gene expression [8]. It has been successfully used to detect gene transcript levels from snap frozen tissue extracts and even from FFPE containing partially fragmented RNA [9-11] although the detection rate is lower as indicated for example by invariably higher C_T _values in the latter [12-16].

**Figure 1:**
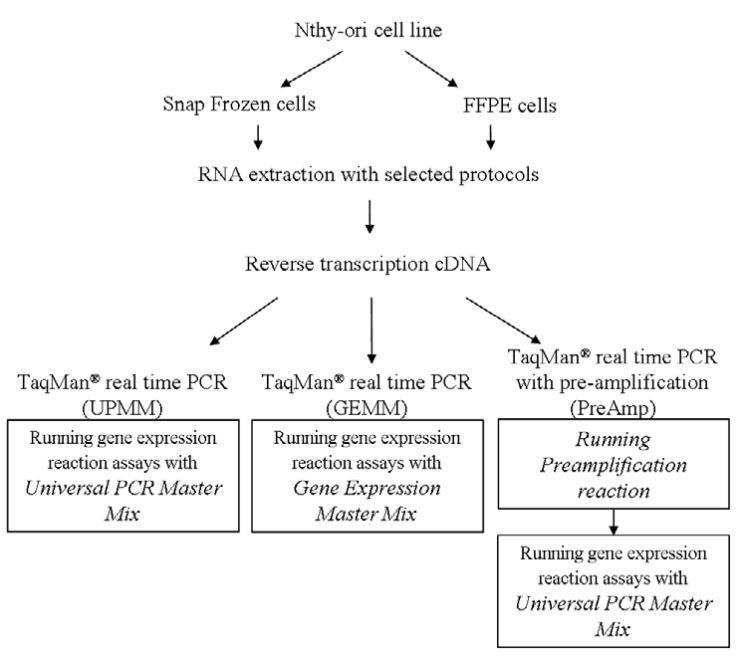
**A schematic representation of the experimental procedures**. Normal thyroid cell lines were split into two aliquots: One was snap frozen, the other formalin fixed and paraffin embedded. RNA was extracted using selected protocols and was then reverse transcribed into cDNA followed by different TaqMan^® ^QRT-PCR analysis.

In this study we examined and optimized selected RNA extraction protocols, including Stratagene Absolutely RNA^® ^FFPE Kit and Ambion RecoverAll™ Total Nucleic Acid Isolation Kit, by comparison of parallel extracts from FFPE and snap frozen cell preparations using a cell line model (Figure 1). We further analyzed these extracts using different TaqMan^® ^protocols, including two types of TaqMan^® ^Master Mix (Universal PCR Master Mix (UPMM) and Gene Expression Master Mix (GEMM)) and newly developed TaqMan^® ^with pre-amplification method (PreAmp), with a panel of assays over a range of amplicon sizes.

## Results

### RNA extraction

RNA quantity was assessed spectrophotometrically using NanoDrop^® ^ND-1000 Spectrophotometer (Wilmington, USA), which showed that the yields from snap frozen extracts were greater than those from FFPE when RNA was extracted from identical numbers of cells using all the protocols examined (Stratagene Absolutely RNA^® ^FFPE Kit, Ambion RecoverAll™ Total Nucleic Acid Isolation Kit, Gentra Purescript^® ^RNA Purification Kit and Invitrogen Trizol^® ^Reagent). In comparison of FFPE extracts (Table 1), Ambion gave the highest yields, and column based Stratagene and Ambion protocols produced clean RNA with OD 260/280 ratio greater than 1.8. RNA quality was assessed using Agilent 2100 Bioanalyzer (Agilent technologies, Waldbronn, Germany) and TaqMan^® ^RT-PCR, which showed variations in RNA quality dependant on the protocol used. Stratagene and Ambion RecoverAll™ kits gave superior FFPE RNA results with regard to quality than the others examined.

**Table 1:** RNA yields from FFPE materials. Extraction products were assessed spectrophotometrically using NanoDrop^® ^ND-1000 Spectrophotometer. Three extractions were carried out using each protocol.

	OD Ratio (260/280)	Yields (ng)
Ambion	2.1 +/- 0.1	337 +/- 58
Stratagene	2.1 +/- 0.1	147 +/- 26
Gentra	1.6 +/- 0.1	186 +/- 67
Trizol	1.6 +/- 0.1	108 +/- 72

### Evaluation of modified protocols

**Figure 2:**
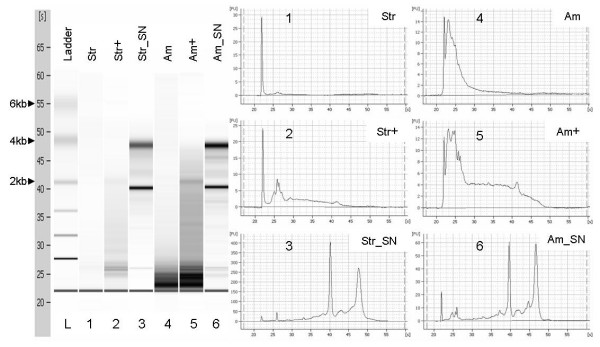
**Qualities of RNA were measured using Agilent 2100 Bioanalyzer**. (Str = Stratagene protocol; Am = Ambion protocol; + = modified with incubation in Proteinase K buffer at 70°C for 20 minutes; and SN = snap frozen.) Modification to the Stratagene and Ambion protocols generated larger fragments (lane 2 and 5) compared to the extracts generated using the original protocols (lane 1 and 4). Each well contained 1 μl of extracted RNA from equal amount of starting materials of 10^5 ^cells.

**Figure 3:**
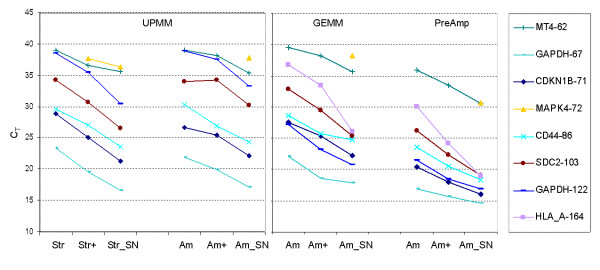
**TaqMan^® ^gene expression pattern using UPMM, GEMM and PreAmp**. (Str = Stratagene protocol; Am = Ambion protocol; + = modified protocols with incubation in Proteinase K buffer at 70°C for 20 minutes; and SN = snap frozen cells. Not all assays produced products, e.g. HLA_A in UPMM.) C_T_s were higher using FFPE extracts compared to snap frozen counterparts when the amount of input RNA was identical. Modification to Stratagene and Ambion protocols produced decreased C_T_s. TaqMan^® ^with UPMM generated higher C_T_s than GEMM and PreAmp when using longer amplicon lengths. This was particularly evident when comparing two GAPDH assays – one 122 bp and the other 67 bp. A mean difference of 17 cycles between small and large assays using UPMM and a mean difference of 4 cycles between small and large assays using GEMM was observed.

Modification to the Stratagene and Ambion protocols generated approximately 25–40% greater yields and larger fragments of RNA (Figure 2) than the standard procedure. Adjustment to Stratagene and Ambion protocols produced decreased C_T_s (e.g. with a mean of 2.95 cycles in GEMM experiment and a mean of 3.14 cycles in PreAmp), indicating the improved quality of RNA extracted from FFPE (Figure 3).

**Figure 4:**
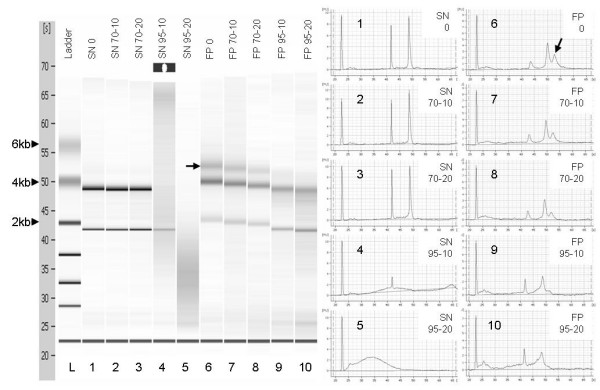
**The effect of different incubation conditions on RNA**. Total RNA was extracted from a large number (2 × 10^6 ^cells) of formalin fixed paraffin embedded cells using Ambion RecoverAll™ kit. Five eluted RNA aliquots from one extraction were subjected to different incubation conditions on a hot rack (lanes 6 to 10). In parallel, five eluted RNA aliquots from one snap frozen extraction were incubated (lane 1 to 5). Each well contained 1 μl of 4 ng/μl RNA in Ambion Elution Solution. Incubation conditions were as follows: 0 = no treatment; 70-10 = 70°C for 10 min; 70-20 = 70°C for 20 min; 95-10 = 95°C for 10 min and 95-20 = 95°C for 20 min. RNA from snap frozen preparations was not degraded at 70°C for 20 min (lane 3), but was degraded at 95°C (lane 4 and 5). RNA from FFPE showed large fragments and cross-linked RNAs which are approximately 5 kb (lane 6 – arrow). It was found that incubation of eluted RNA at 70°C for 20 min is the optimal condition, among these tested, to break up cross-links while not compromising RNA integrity.

A separate experiment using Ambion RecoverAll™ kit enabled extraction of large RNA molecules including cross-linked RNAs (Figure 4). Incubation of eluted RNA at 70°C for 20 minutes was found to be the best condition to disrupt cross-links while not compromising RNA integrity in comparison with other modifications (70°C for 10 min, 95°C for 10 min and 95°C for 20 min).

### Comparison of TaqMan^® ^Universal PCR Master Mix (UPMM), Gene Expression Master Mix (GEMM) and PreAmp

**Figure 5:**
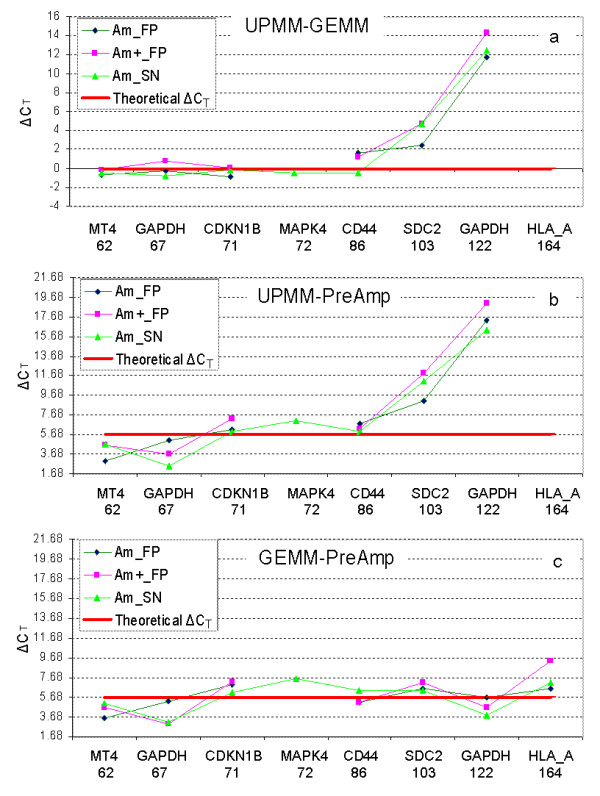
**Comparison of TaqMan^® ^gene expression pattern using ΔC_T _method**. (Am = Ambion protocol; FP = FFPE cells; + = modified protocols with incubation in Proteinase K buffer at 70°C for 20 minutes; and SN = snap frozen cells. Not all assays produced products, e.g. HLA_A in UPMM.) These ΔC_T _data was generated from the C_T_s shown in Figure 3. A Theoretical ΔC_T _was calculated for each chart based on equilibrating the results for any variation in input cDNA. In panel a, ΔC_T _= C_T__UPMM - C_T__GEMM. The Theoretical ΔC_T _of UPMM-GEMM was 0 [= Log_2_(20 ng/20 ng)] given identical input quantities (20 ng) were used in each system. In panel b, ΔC_T _= C_T__UPMM - C_T__PreAmp. In panel c, ΔC_T _= C_T__GEMM - C_T__PreAmp. The Theoretical ΔC_T _in panel b and c was 5.68 [= Log_2_(1024 ng/20 ng)], which was calculated based on an input of 1024 ng of cDNA for the TaqMan^® ^real time PCR component of pre-amplification process. This quantity was generated from an initial 1 ng subjected to 10 cycles of pre-amplification with a 100% efficiency and no bias introduced from the PreAmp. The relevant Theoretical ΔC_T _is plotted on each chart as a reference point for measuring the actual detected ΔC_T _against the theoretically optimal ΔC_T_. The benefit of GEMM over UPMM was evident as amplicon size increased (Panel a). A comparison of UPMM and PreAmp showed a similar pattern (Panel b). However, PreAmp results generally correlated with GEMM regardless of amplicon size for the series of 8 assays analysed (Panel c).

C_T _values were also dependent on the type of TaqMan^® ^PCR Master Mix that was used (Figure 3 and Figure 5). TaqMan^® ^with UPMM generated higher C_T_s for long amplicons than GEMM. This was observed by a comparison of two GAPDH assays – one 122 bp and the other 67 bp. There was a mean threshold detection difference of 17 cycles using UPMM and a mean difference of 4 cycles using GEMM with RNA template extracted using Ambion RecoverAll™ kit (Figure 3). In addition, HLA_A is the largest amplicon (164 bp) which produced no product with UPMM, however, GEMM and PreAmp both allowed the detection of this amplicon size (Figure 3). GEMM improved the C_T _values for long amplicons (over 100 bp) compared with UPMM (Figure 5-a). When the ΔC_T_s were compared to Theoretical ΔC_T_s, PreAmp results generally correlated with GEMM for all the 8 assays analyzed (Figure 5-c).

### Evaluation of TaqMan^® ^PreAmp using CDKN1B

**Figure 6:**
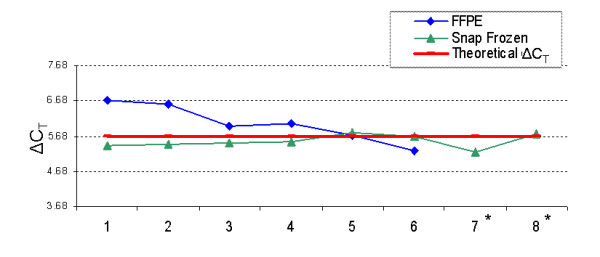
**Comparison of TaqMan^® ^real time PCR with or without PreAmp using CDKN1B assay on extracts produced by different extraction protocols**. (1 and 2 = Stratagene extracts; 3 and 4 = Ambion extracts; 5 and 6 = Gentra extracts; 7 and 8 = Trizol extracts; * = No amplification was achieved from FFPE) ΔC_T _= C_T _(no_PreAmp) - C_T _(with_PreAmp). The Theoretical ΔC_T _was 5.68 [= Log_2_(1024 ng/20 ng)] which was calculated based on an input of 1024 ng of cDNA for the TaqMan^® ^real time PCR component of pre-amplification process and 20 ng of cDNA for the TaqMan^® ^without pre-amplification. Ideally, ΔC_T _is in a range between Theoretical ΔC_T _+/- 1 which was achieved in this experiment indicating there is no bias in the PreAmp.

TaqMan^® ^PreAmp analysis consistently achieved decreased C_T _values in both snap frozen and FFPE aliquots compared with TaqMan^® ^without pre-amplification step (Figure 5-b, c). A good correlation between PreAmp and UPMM was observed using CDKN1B to analyze RNA extracted with different protocols (Figure 6), suggesting that PreAmp does not introduce any bias into the reaction.

### Comparison of C_T _difference between FFPE and snap frozen cells

**Figure 7:**
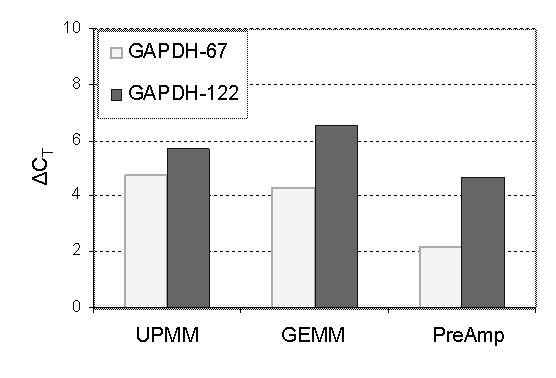
**Analysis of GAPDH using two sizes of amplicon showing the difference of C_T_s between FFPE and snap frozen counterparts**. ΔC_T _= C_T _(FFPE) - C_T _(Snap-Frozen). The smaller amplicon size of GAPDH (67 bp) generated lower ΔC_T_s than the larger amplicon size of 122 bp indicating that smaller amplicon sizes produced more comparable C_T_s in snap frozen and FFPE samples.

TaqMan^® ^analysis over all assays showed C_T_s to be 2 to 11 cycles higher when using FFPE extracts compared to snap frozen counterparts when the amounts of input RNA were identical (Figure 3). Focussing on an analysis of GAPDH using two assays with different amplicon sizes revealed the smaller amplicon assay (67 bp) produced C_T_s from snap frozen and FFPE samples that were closer together (Figure 7).

## Discussion

RNA analysis has generally been performed on snap-frozen or fresh materials, using variable techniques including microarray, northern blotting and RT-PCR. However, a constant challenge has been robust and reliable analysis of gene expression in archival tissues. This application has been frustrated by poor RNA yields, small sizes of extracted fragments and low levels of detectable RNA in the extracts [12]. In this study we established a cell line model to test modified extraction protocols and quantify differences in performance using a panel of QRT-PCR assays on FFPE compared with parallel snap-frozen cell preparations using TaqMan^® ^methodology. We found that our modified extraction method and TaqMan^® ^PreAmp enhanced expression analysis from FFPE cells optimally.

### RNA and FFPE

Generally, RNA extracted from FFPE materials is fragmented and chemically modified. Fragmentation occurs possibly since tissues are surgically removed, and it continuously occurs during fixation and preservation. Experimental design was such that equal numbers of cells were processed for FFPE cell block construction and snap freezing for comparative purposes. By using cells fixed under controlled conditions possible variables due to the effects of storage on RNA degradation were negated, thus revealing the true impact of formalin fixation on RNA quality. We extracted intact RNA fragments in a separate experiment from pellets with a large number of formalin fixed paraffin embedded normal thyroid cells (Figure 4), which was consistent with a finding by Scicchitana et al [17]. In that study, intact RNA molecules were extracted from newly formalin fixed paraffin embedded human bone marrow stromal cells suggesting that RNA degradation could be a minor problem for recently fixed FFPE in some types of cells. Our study showed that the differences in RNA quality between the small and large fixed pellets (Figure 2 and Figure 4) might be due to the fact that the majority of cells in the centre of the large pellet were remained alive during construction of the large solid cell pellet. This could limit the access of RNases to intracellular mRNA because degradation will not fully occur until the fixation process begins resulting to the intact mRNA being maintained. Unfortunately, intact mRNA is not generally obtained in majority of fixed tissues or cell lines, and RNA extracted from FFPE is normally degraded to fewer than 300 bp.

Figure 4 displays the degree of RNA modification in FFPE cells caused by methylol groups during formalin fixation. Masuda and colleagues [18] suggested that formaldehyde reacts with RNA forming an N-methylol followed by an electrophilic attack to form a methylene bridge between amino groups. Interestingly, our results show a clear large RNA band, approximately 5 kb in length (Figure 4-lane 6), which is hypothetically the cross-linked RNA. The other two bands visualized were also larger than that extracted from snap frozen cells indicating the modification of RNA in FFPE. Furthermore, with incubation at high temperature, the large RNA band (~5 kb) was removed, and the sizes of the other two bands became closer to 18 s and 28 s of the snap frozen extracts. This is in agreement with the results of a mass spectrometric analysis that was carried out by Masuda group, suggesting methylol modification is reversible by heating [18,19]. However, a balance must be achieved between breaking cross-links while not contributing to degradation of labile RNA. Our results showed that incubation at 70°C for 20 minutes was the optimal condition, among those tested, to de-modify cross-linked RNA while maintaining RNA integrity (Figure 4).

### Extraction protocols

A prerequisite for gene expression studies in formalin-fixed tissue samples was the establishment of a reliable and reproducible extraction method to provide detectable RNAs for subsequent analysis [1]. The quality of extracted RNA can be variable among different extraction methods due to several factors, such as the contamination of RNases, proteins and genomic DNA [20]. Our results showed that proteinase K digestion based protocols followed by on column DNase digestion and RNA elution produced good detectable RNA of FFPE.

Proteinase K digestion has also been demonstrated by many laboratories to be critical in RNA extraction protocol [1,3,21,22] possibly because of two functions. On one hand, it can degrade proteins that are covalently cross-linked with each other and nucleic acid to release RNA from the matrix, thereby allowing efficient RNA extraction from FFPE materials [21]. On the other, it can inactivate RNases that tend to be stable and do not require cofactors to function. Thus its activity avoids any potential reactivation of RNase during reversal of fixation in aqueous buffers [1].

Our data demonstrates that incubation in proteinase K buffer at 70°C for 20 minutes facilitated the disruption of cross-links, resulting in improved quantity and quality of RNA. The RNA extracted using the modified protocols (with the incubation) enhanced detection by a mean of 3 cycles (Figure 3), which is equivalent to 8 fold increased sensitivity. It is most likely that the additional incubation step removed the remaining cross-links between RNA and protein leading to the longer RNA molecules being extracted [23]. In addition, this incubation denatured proteinase K, thus avoided its further damage to RNA in the following purification procedure, which suggests the necessity of a heating (at 70°C for 20 min) application in any proteinase K based extraction of FFPE samples.

### TaqMan^® ^PreAmp

TaqMan^® ^QRT-PCR technique is based on the 5' nuclease activity of Taq DNA polymerase and involves cleavage of a specific fluorogenic hybridization probe that is flanked by PCR primers [24,25]. Because of the small target size, many laboratories have demonstrated that it is possible to measure gene expression levels using FFPE tissues as a source of mRNA [3]. Still, it seems to be problematic to perform large scale of analysis on FFPE because of the high C_T_s and limited concentration of extracts [14,15].

We employed a novel TaqMan^® ^PreAmp technique which we found to be a practical solution to decrease C_T _values, and in particular suitable in our hands to generate real-time PCR results from limited amounts of input RNA, such as extracted from laser captured microdissected material [26]. The principle of TaqMan^® ^PreAmp technique is to amplify target cDNA prior to real-time TaqMan^® ^PCR analysis. Briefly, cDNA is synthesized from total RNA by use of random priming. The cDNA for the specific target assays is then amplified by pre-amplification reaction using pooled gene-specific primers to increase the number of targeted copies. The pre-amplification product is diluted and finally analyzed by real-time TaqMan^® ^PCR using single assay containing one pair of gene-specific primers and probe.

The TaqMan^® ^PreAmp technique addresses the challenge faced by researchers working with rare or precious samples, which only limited RNA could be extracted from, to perform gene expression analyses using real-time QRT-PCR. The simple process enables the user to perform uniform amplification from as little as one nanogram of cDNA (which was used in this study) or alternatively conduct up to 200 real-time PCR reactions per pre-amplification reaction without compromising the available sample material. Our results demonstrated that TaqMan^® ^PreAmp overcame the difficulties usually caused by low yields of RNA extraction from FFPE.

### Influence of TaqMan^® ^Master Mix and amplicon length

This study demonstrated that the sensitivity of TaqMan^® ^detection was influenced by choice of Master Mix and amplicon length in assay design. We evaluated UPMM and GEMM which showed similar sensitivity for short amplicons (less than 90 bp), while GEMM displayed better sensitivity for longer amplicons (over 100 bp) compared with UPMM in parallel snap frozen and FFPE extracts (Figure 5). This effect was dissipated when UPMM was used after pre-amplification, possibly because of the increased copy number of template available or because of the composition of the PreAmp MasterMix which is closer in components to GEMM than UPMM. Reagents can have a significant effect on assay reproducibility [27] due to some parameters such as different polymerases sensitivity [28], primer binding efficiency and the concentration of Mg^2+ ^[29]. Karrer et al. described the Monte Carlo effect using plant material suggesting that the PCR reproducibility could be limited when the number of available templates is low. Increased concentrations of Mg^2+ ^reduced PCR variation possibly by allowing a higher proportion of annealed primers extended by the more active polymerase [29].

Our data corroborated the observation that amplicon size is crucial in designing assays to analyze gene expression levels not only using FFPE extracts but also using RNAs with high integrity [12,15]. Many researchers have found that short amplicons generated lower C_T_s than longer amplicons on analysis of the same gene in FFPE [30,31]. Data generated in this study evaluating snap frozen samples using two sizes of GAPDH (Figure 3) demonstrated GAPDH-67 generated a reduction in C_T _by 2 – 3 cycles over GAPDH-122 in both GEMM and PreAmp experiments, demonstrating that smaller amplicons give more consistent results [32].

In addition, we found C_T_s between FFPE and snap frozen were closer for small amplicons than that for large amplicons (Figure 7). For example, the analysis of GAPDH using a target amplicon of 67 bp displayed C_T_s 1 – 2.5 cycles closer between FFPE and snap frozen than the same experiment using the longer amplicon size of 122 bp. We further measured the amplification efficiency associated with these two sizes of GAPDH assays using a broad dilution range (5 Log_10_s) which showed closed efficiencies with 99.98% for GAPDH-67 and 99.92% for GAPDH-122 (data not shown). This measurement eliminates non-specific amplification that could contribute to decreased amplification efficiency of the true target. Therefore, it seems reasonable to conclude that the shift of efficiency detected from frozen to fixed material (Figure 7) is due to degradation of RNA in FFPE, and logical to extrapolate that the longer an amplicon is, the more likely its template will be degraded in extracted RNA. As a general rule, house-keeping genes or normalising assays should have amplicon sizes that match the size of the target whose expression is to be measured [3] and amplicons less than 100 bp should be employed in gene expression studies using FFPE materials.

## Conclusion

We evaluated the effect of modifying recommended extraction protocols to reproducibly produce RNA that may be successfully amplified using QRT-PCR. We have found the TaqMan^® ^PreAmp system to be a robust and practical solution to limited quantities of RNA and have demonstrated comparable results in matched FFPE and snap frozen preparations providing proof of principle that this method may reliably be utilised in the context of multiple expression analyses from individual FFPE samples.

## Methods

### Cell culture and formalin fixation

Nthy-ori 3-1 (ECACC, Wiltshire, UK) is a normal thyroid follicular epithelial cell line derived from adult thyroid tissue that has been transfected with a plasmid encoding for the SV40 large T gene [33].

This cell line was grown to confluence in a humidified atmosphere containing 5% CO_2 _at 37°C in the following plating medium: RPMI 1640 with 2 mM L-glutamine, 10% Foetal calf serum (FCS), Penicillin (100 U/ml) and Streptomycin (100 μg/ml). Tripsinized cells were counted with a hemocytometer. Approximately 1 × 10^5 ^suspended cells were aliquot and were pelleted (a) snap frozen and (b) formalin fixed and paraffin embedded into a cell block. When formalin fixation was required, a cohesive solid cell pellet was constructed using 20% agar [23]. The cells were centrifuged in an eppendorf tube, and the supernatant was removed using a pipette. Approximately 30 μl of pre-warmed agar (60°C) was added to each tube. The solid cell pellet was formed within a few seconds. Cell blocks were placed in 10% buffered formalin at room temperature for 5 hours followed by tissue processing on a Tissue-Tek^® ^V.I.P.TM tissue processor for 8 hours comprising: 10% buffered formalin fixation (4 hours at 37°C), 60% ethanol (20 minutes at 37°C), 80% ethanol (20 minutes at 37°C), 100% ethanol (20 minutes at 37°C), xylene (40 minutes at 37°C) and paraffin (80 minutes at 60°C). The pellets were subsequently paraffin embedded.

### RNA extraction protocols

RNA extraction was performed using 4 protocols (Table 2): Ambion RecoverAll™ Total Nucleic Acid Isolation Kit, Stratagene Absolutely RNA^® ^FFPE Kit, Gentra Purescript^® ^RNA Purification Kit, and Invitrogen Trizol^® ^Reagent, in addition to modified Stratagene and Ambion protocols. Apart from deparaffinazition, RNA was extracted from snap frozen and FFPE cells in parallel according to manufacturer's protocols including proteinase K digestion (not included in Trizol^® ^protocol), RNA isolation and elution or hydration procedures. The modification in Stratagene and Ambion protocols involved incubation at 70°C for 20 minutes after the recommended proteinase K digestion.

**Table 2:** Overview of the RNA extraction protocols. Four RNA extraction protocols are Ambion RecoverAll™ Total Nucleic Acid Isolation Kit, Stratagene Absolutely RNA^® ^FFPE Kit, Gentra Purescript^® ^RNA Purification Kit and Invitrogen Trizol^® ^Reagent. (RT. = Room temperature)

	**Ambion**	**Stratagene**	**Gentra**	**Trizol**
De-paraffiniztion	Xylene 50°C for 3 min Ethanol washes	Deparaffin – Reagent RT. for 10 min Ethanol washes	Xylene RT. for 5 min Ethanol washes	Xylene RT. for 5 min Ethanol washes
Proteinase K digestion	4 μl Protease in 400 μl digestion buffer (final concentration unknown) 50°C for 3 hours	10 μl × 20 mg/ml proteinaseK in 100 μl digestion buffer (final concentration 1.82 μg/ul) 55°C overnight	1.5 μl × 20 mg/ml proteinaseK in 300 μl cell lysis solution (final concentration 0.10 μg/ul) 55°C overnight	--
RNA isolation	Add Isolation Additive Add Ethanol Pass the mixture through a Filter Cartridge Buffer washes On column DNase digestion	Add β-ME Pass the mixture through a prefilter spin cup Add Ethanol Pass the mixture through a RNA binding cup Buffer washes On column DNase digestion	Add protein-DNA precipitation solution Separate the supernatant Add Isopropanol and Glycogen -20°C for 1 hour Pellet RNA and Ethanol wash	Homogenize in 1 ml Trizol Reagent Add Chloroform Separate the aqueous phase Add Isopropanol and Glycogen RT. for 10 min Pellet RNA and Ethanol wash
RNA collection	Elution buffer 30 μl × 2	Elution buffer 30 μl	RNA hydration solution 30 μl	H_2_O 30 μl

RNA quantity was assessed using the NanoDrop^® ^ND-1000 Spectrophotometer (Wilmington, USA), and the quality was measured using the RNA 6000 Pico LabChip^® ^Kit on an Agilent 2100 Bioanalyser (Agilent technologies, Waldbronn, Germany).

### TaqMan^® ^gene expression assays

Seven TaqMan^® ^Gene Expression Assays (P/N: 4331182, Applied Biosystems, CA, USA) and one custom designed assay (GAPDH-67) were utilised in this study with a range of amplicon sizes from 62 to 164 (Table 3). The extracted RNA was reverse transcribed into cDNA and were then quantified using TaqMan^® ^real time PCR with or without PreAmp procedure.

**Table 3:** Eight of TaqMan^® ^Gene Expression Assays. Seven of these assays were inventoried by Applied Biosystems (P/N: 4331182, Applied Biosystems, CA, USA) and one was a designed assay (GAPDH-67 Forward primer: CAT CCA TGA CAA CTT TGG TAT CGT; Reverse primer: GGG TGG CAG TGA TGG CAT; Probe: ACT CAT GAC CAC AGT CC).

Gene Symbol – Amplicon Length	Gene Name	Assay ID
MT4 – 62	metallothionein IV	Hs00262914_m1
GAPDH – 67	glyceraldehyde-3-phosphate dehydrogenase	Designed
CDKN1B – 71	cyclin-dependent kinase inhibitor 1B (p27, Kip1)	Hs00153277_m1
MAPK4 – 72	mitogen-activated protein kinase 4	Hs00177074_m1
CD44 – 86	CD44 molecule (Indian blood group)	Hs00153304_m1
SDC2 – 103	syndecan 2 (heparan sulfate proteoglycan 1, cell surface-associated, fibroglycan)	Hs00299807_m1
GAPDH – 122	glyceraldehyde-3-phosphate dehydrogenase	Hs99999905_m1
HLA-A – 164	major histocompatibility complex, class I, A	Hs00740413_g1

#### Reverse transcription (RT)

Applied Biosystems High-Capacity cDNA Archive Kit (P/N: 4322171, Applied Biosystems) was used following manufacturer's protocol for reverse transcription. Each RT reaction contained 50 μl of 8 ng/μl total RNA, 10 μl of 10× RT buffer, 4 μl of 25× dNTP mixture, 10 μl of 10× Random Primers, 5 μl of MultiScribe RT (50 U/μl) and 21 μl of RNase-free water. The 100 μl reactions were incubated in an Applied Biosystems Thermocycler for 10 min at 25°C, 2 hours at 37°C and then held at 4°C.

#### TaqMan^® ^real time PCR

For the Real-time PCR step, amplification was carried out on the Applied Biosystems 7000 Sequence Detection System. Two types of TaqMan^® ^Master Mix were employed in this procedure: TaqMan^® ^Universal PCR Master Mix with UNG (P/N 4304437, Applied Biosystems) or TaqMan^® ^Gene Expression Master Mix (P/N 4370048, Applied Biosystems). The 20 μl PCR reaction included 10 μl of 2× TaqMan^® ^Master Mix, 5 μl of 4× TaqMan^® ^Gene expression Assay (P/N 4331182, Applied Biosystems) and 5 μl of cDNA (RT product 4 ng/μl). The reactions were incubated in a 96-well optical plate at 50°C for 2 min, at 95°C for 10 min, following by 40 cycles of 95°C for 15 seconds and 60°C for 1 min. The real-time PCRs for each assay were run in triplicate.

#### TaqMan^® ^real time PCR with PreAmp

The preamplification was performed using TaqMan^® ^PreAmp Master Mix Kit protocol (P/N 4366128, Applied Biosystems). The pooled assay mix was prepared by combining 8 of 20× TaqMan^® ^Gene Expression Assays into a single tube and using 1× TE buffer to dilute the pooled assays to a final concentration of 0.2×. The 40 μl of preamplification reaction included 20 μl of 2× TaqMan^® ^PreAmp Master Mix, 10 μl of 0.2× pooled assay mix and 10 μl of 4 ng/μl cDNA sample. The reactions were incubated in an Applied Biosystems Thermocycler for 10 min at 95°C following by 10 cycles of 95°C for 15 seconds and 60°C for 4 min and then held at 4°C. The concentration of the preamplification product was 2^10 ^ng/μl (in theory) which was then 1:5 diluted and analyzed by TaqMan^® ^real time PCR using TaqMan^® ^Universal PCR Master Mix following the procedures described.

### Data analysis

Replicates were omitted if C_T _standard deviation was greater than 1.5 in the triplicate. All the data were collected in Excel form. The formulas used to generate the figures are as below:

1. ΔC_T _(A-B) = C_T _X_mean(A) - C_T _X_mean(B)

2. Theoretical ΔC_T _= Log_2_(Amount cDNA in A/Amount of cDNA in B)

## Authors' contributions

JL performed the RNA extraction and TaqMan^® ^analysis and wrote original and final versions of the manuscript. PS, SC helped with the extraction and TaqMan^® ^analysis. PS, RF, KD helped draft the manuscript. KD, SA carried out cell culture. MP, SG helped with the analysis of the data. JOL and OS conceived the study and helped write the original and final versions of this manuscript. All authors read and approved the final manuscript.
